# The Genetic Basis of Anthocyanin Acylation in North American Grapes (*Vitis* spp.)

**DOI:** 10.3390/genes12121962

**Published:** 2021-12-09

**Authors:** Avinash Karn, Luis Diaz-Garcia, Noam Reshef, Cheng Zou, David C. Manns, Lance Cadle-Davidson, Anna Katharine Mansfield, Bruce I. Reisch, Gavin L. Sacks

**Affiliations:** 1 School of Integrative Plant Science, Cornell AgriTech, Cornell University, Geneva, NY 14456, USA; akarn@cornell.edu (A.K.); lance.cadledavidson@usda.gov (L.C.-D.); bruce.reisch@cornell.edu (B.I.R.); 2 Instituto Nacional de Investigaciones Forestales, Agrícolas y Pecuarias, Campo Experimental Pabellón, Aguascalientes 20676, Mexico; 3 Department of Food Science, Cornell University, Ithaca, NY 14853, USA; gls9@cornell.edu; 4 BRC Bioinformatics Facility, Institute of Biotechnology, Cornell University, Ithaca, NY 14853, USA; cz355@cornell.edu; 5 Department of Food Science, Cornell AgriTech, Cornell University, Geneva, NY 14456, USA; dcm38@cornell.edu (D.C.M.); akm87@cornell.edu (A.K.M.); 6 USDA-Agricultural Research Service, Grape Genetics Research Unit, Geneva, NY 14456, USA

**Keywords:** coumaroylated anthocyanins, QTL mapping, rhAmpSeq, fruit chemistry, wine pigments

## Abstract

Hydroxycinnamylated anthocyanins (or simply ‘acylated anthocyanins’) increase color stability in grape products, such as wine. Several genes that are relevant for anthocyanin acylation in grapes have been previously described; however, control of the degree of acylation in grapes is complicated by the lack of genetic markers quantitatively associated with this trait. To characterize the genetic basis of anthocyanin acylation in grapevine, we analyzed the acylation ratio in two closely related biparental families, *Vitis rupestris* B38 × ‘Horizon’ and ‘Horizon’ × Illinois 547-1, for 2 and 3 years, respectively. The acylation ratio followed a bimodal and skewed distribution in both families, with repeatability estimates larger than 0.84. Quantitative trait locus (QTL) mapping with amplicon-based markers (rhAmpSeq) identified a strong QTL from ‘Horizon’ on chromosome 3, near 15.85 Mb in both families and across years, explaining up to 85.2% of the phenotypic variance. Multiple candidate genes were identified in the 14.85–17.95 Mb interval, in particular, three copies of a gene encoding an acetyl-CoA-benzylalcohol acetyltransferase-like protein within the two most strongly associated markers. Additional population-specific QTLs were found in chromosomes 9, 10, 15, and 16; however, no candidate genes were described. The rhAmpSeq markers reported here, which were previously shown to be highly transferable among the *Vitis* genus, could be immediately implemented in current grapevine breeding efforts to control the degree of anthocyanin acylation and improve the quality of grapes and their products.

## 1. Introduction

Anthocyanin pigments are widespread in the plant kingdom, including grapevines (*Vitis* spp.), where they contribute to the coloration of red/black grape berries and resulting products such as juices and wines [1]. Anthocyanins consist of a flavonoid aglycone (an anthocyanidin, e.g., malvidin) covalently linked to one or more sugars [2]. Anthocyanins may be further modified by the acylation of their sugar group(s) by one or more organic acid moieties. The dominant acylated forms in most *V. vinifera* cultivars are derivatives of hydroxycinnamic acids (e.g., anthocyanidin-3-*O*-ferulylglucosides, anthocyanidin-3-*O*-coumarylglucosides) [1]. These hydroxycinnamylated anthocyanins, referred to in this paper simply as ‘acylated anthocyanins’, are of interest because of their enhanced color stability relative to non-acylated anthocyanins in typical food or beverage matrices [3]. For example, as compared to non-acylated analogs, acylated anthocyanins show little change in their absorption profiles over the pH range 3–5 and are less susceptible to thermal hydrolysis [4], purportedly due to intramolecular stacking between the flavonoid and hydroxycinnamyl rings.

The proportion of anthocyanins that exist in acylated forms can vary widely among grape cultivars. For example, a survey of 50 Spanish *V. vinifera* cultivars reported that the acylated anthocyanin fraction (specifically, the coumarylated fraction) varied from 4.2% to 53.5% [5], and some black-fruited cultivars such as Pinot noir are reported to have no detectable acylated anthocyanins. Similar variation in the degree of acylation has been reported among wild *Vitis* accessions [6]. DNA markers predicting acylation could be of interest to breeders, e.g., to select for grapes with high proportions of acylated species to achieve greater color stability in grape-derived products or to select for lower acylation to increase anthocyanin bioavailability [7,8] and consequent health benefits [9,10,11].

The biosynthetic pathway of anthocyanins in grapes and other plants is well understood [2], and several markers associated with anthocyanin-related traits have been discovered through mapping studies. MYB transcription factors are critical for regulating total anthocyanin production [12]. Studies with transgenic grapes have demonstrated that VvMYBA is a positive regulator of the later stages of anthocyanin biosynthesis and modification, including the formation of acylated anthocyanins by an anthocyanin acyltransferase (VvAT) [13]. Moreover, work led by Massonnet et al. 2017 [14] identified that the expression of VvAT, as well as other anthocyanin biosynthesis-related genes, was unique to red grape cultivars. QTL analysis of a ‘Syrah’ × Pinot noir mapping population identified several minor QTLs for anthocyanin acylation, including two VvAT genes [15]. However, no further QTL mapping studies have associated VvAT with the presence/absence of acylated anthocyanins in *Vitis*.

To identify novel markers for anthocyanin acylation, we quantified the anthocyanin acylation ratios (acylated anthocyanins relative to the total anthocyanin content) in two genetically related *Vitis* biparental populations, *V. rupestris* B38 × ‘Horizon’ and ‘Horizon’ × Illinois 547-1. These populations segregated for the degree of anthocyanin acylation during 2 and 3 consecutive years, respectively. A set of 2000 amplicon-based markers (rhAmpSeq) were applied to find a stable major QTL on chromosome 3. An analysis of genomic regions harboring this marker-trait association revealed acyltransferase candidate genes regulating the presence/absence of anthocyanin acylation in grapes. Moreover, our highly transferable rhAmpSeq markers linked to anthocyanin acylation could be rapidly adopted in current breeding programs for the selection of grape materials with improved fruit quality.

## 2. Materials and Methods

### 2.1. Plant Material and Sampling

Two F_1_ grapevine families, *V. rupestris* B38 × ‘Horizon’ and ‘Horizon’ × Illinois 547-1 (material used as female showed first), were grown without replication (single vines of each genotype; additional vines of each parent) in Geneva, New York. ‘Horizon’ (white grape cultivar) is a complex hybrid and includes ancestry from *V. vinifera*, *V. labrusca*, *V. aestivalis*, *and V. rupestris* [16]. Illinois 547-1 (male with no fruit production) resulted from a cross between *V. rupestris* B38 × *V. cinerea* B9; therefore, *V. rupestris* B38 (the only parent accumulating anthocyanins) is a parent in one family and a grandparent in the other. Fruit sampling and analysis were conducted in 2012 and 2013 for *V. rupestris* B38 × ‘Horizon’, and in 2011–2013 for ‘Horizon’ × Illinois 547-1. The progeny consisted of 118 and 116 vines in *V. rupestris* B38 × ‘Horizon’ and 93, 162, and 142 vines in ‘Horizon’ × Illinois 547-1, in each year, respectively. For each genotype, 150 g of fruit were sampled at maturity, based on fruit total soluble solid content (°Brix). TSS means and standard deviation at maturity in the families were 22.7 ± 1.9 and 18.4 ± 1.4 in 2012 and 2013 in *V. rupestris* B38 × ‘Horizon’ and 17.1 ± 2, 21.4 ± 2.3, and 20.1 ± 2.1 in 2011–2013 in ‘Horizon’ × Illinois 547-1. In addition, in 2013, ‘Horizon’ × Illinois 547-1 fruits were analyzed at an earlier phenological stage, i.e., mean TSS level of 16.5 ± 2.1, to study the effect of ripening stage on the degree of acylation. Sampled fruit was transported in cooler boxes and stored at −20 °C before shipping to the Cornell Ithaca campus for phenotyping.

### 2.2. Phenotyping and Data Analysis

Frozen berry samples were destemmed, partially thawed, and homogenized in a 250 mL stainless steel Waring blender (Stamford, CT, USA) at medium speed for 30 s and then at high speed for 30 s. A 10 g portion of berry slurry was transferred to a 15 mL centrifuge tube and frozen at −20 °C. Prior to analysis, samples were thawed, equilibrated to room temperature, and centrifuged (5 min, 10,000× *g*). An anthocyanin fraction was then isolated by solid-phase extraction (SPE) fractionation based on the protocol of Manns et al. 2012. HPLC analyses were performed on an Agilent Model 1260 Infinity series consisting of an in-line vacuum degasser, autosampler, binary pump, diode array detector, and thermostatted column compartment controlled via Chemstation software (Version 3.04.02SP1 with spectral pack). All solvents, including methanol, acetonitrile, ethyl acetate, and formic acid (Thermo Fisher Scientific, Waltham, MA, USA), were high-performance liquid chromatography (HPLC) grade. Anthocyanin HPLC analyses were based on the method described by Manns et al. 2012. Separations were performed on a Kinetex C18 column (100 mm × 4.6 mm, 2.6 μm particle size) fitted with a KrudKatcher guard filter (Phenomenex, Torrance, CA, USA). Anthocyanin identification was based on commercial standards where possible and otherwise based on characterized color extracts, retention times, and UV absorption characteristics (i.e., appearance of a ~320 nm shoulder in hydroxycinnamic-acylated forms) [17]. Quantification was based on malvidin-3-glucoside and malvidin-3,5-diglucoside standards (Extrasynthese; Genay, France) as measures of mono- and diglucoside equivalents, respectively. Negligible concentrations of acetylated anthocyanins were detected in the populations and were ignored, and “acylated anthocyanins” were taken to refer only to hydroxycinnamic-acylated anthocyanins. Cumulative concentrations of total anthocyanins and acylated anthocyanins were then calculated, along with the ratio of acylated to total anthocyanins (Supplementary File S1).

Broad-sense heritability was estimated for each population according to REML (restricted maximum likelihood) variance components using the following equation: H^2^ = (σ_G_^2^)/(σ_G_^2^ + σ_ε_^2^/*n*), where σ_G_^2^ and σ_ε_^2^ are the variances of the genetic and residuals variance, respectively, and *n* is the number of experiments over the years, respectively.

### 2.3. rhAmpSeq Genotyping and Quality Control

DNA from individuals was extracted using modified Qiagen DNeasy columns with 2.7% PVP-40 added to the extraction buffer, as previously described [18]. rhAmpSeq local haplotype sequencing targeting the core *Vitis* genome was conducted as described in the work of [19,20]. rhAmpSeq markers targeted 250 bp regions evenly distributed across the genome; because there could be multiple polymorphisms (SNPs and indels) per region, each marker represented up to four alleles in the F1 populations studied here. To identify haplotype variants, rhAmpSeq marker data were analyzed using a custom Perl script (https://github.com/avinashkarn/analyze_amplicon/blob/master/analyze_amplicon.pl; (accessed on 2 April 2021)), which was further converted to a variant calling format (VCF) file using the haplotype_to_VCF.pl perl script (https://github.com/avinashkarn/analyze_amplicon/blob/master/haplotype_to_VCF.pl (accessed on 2 April 2021)).

Multidimensional scaling (MDS) and Mendelian error detection were performed as quality controls (Supplementary Files S2 and S3) to identify vines with genotyping errors due to self-pollination, pollen contamination, or mislabeling as described elsewhere [21]. An identical by state (IBS) kinship matrix computed in TASSEL 5 [22] was used to conduct the MDS analysis, which was further visualized using the R ggplot2 package [23]. Similarly, the Mendelian error detection analysis was conducted using the Mendelian plugin in bcftools [24].

### 2.4. Genetic Map Construction

A genetic map was constructed in Lep-MAP3 v. 0.2 [25] using the VCF format files and the pedigree information of each vine in the two mapping populations (*V. rupestris* B38 × ‘Horizon’ and ‘Horizon’ × Illinois 547-1, Supplementary Files S4 and S5). The following Lep-MAP3 modules and steps were used to construct the genetic maps: (1) ParentCall2 module of Lep-MAP3 was used to call parental genotypes; (2) Filtering2 module was used to filter distorted markers based on a χ^2^(chi-squared, *p*-value > 1 × 10^−6^) test and monomorphism (non-segregating); (3) SeparateChromosomes2 module was used to split the marker into linkage groups (LG); (4) OrderMarkers2 module was used to order the markers within each LG using 20 iterations per group, computing parental (sex-specific) and sex-averaged genetic distances. The marker order of the genetic maps was evaluated for the consistency, genome organization, and structural variation by correlating with their physical coordinates on the *V. vinifera* PN40024 reference genome (version 12X.v2; [26]). Finally, the phased output data from the OrderMarkers2 step were converted into 4-way phased genotype data using the map2genotypes.awk script.

### 2.5. QTL Analysis

Sex-averaged genetic maps were used for QTL mapping in the R package qtl [27]. The genotype probabilities were calculated using calc.genoprob with step = 0 (probabilities were calculated only at the marker locations) and assumed genotyping error rate of 1.0 × 10^−4^. Consistently with the observed binomial distribution on anthocyanin acylation suggesting a single major gene, we tested normal and binomial single-QTL models on acylation ratios and total acylated anthocyanins using the function scanone. Significance thresholds were defined based on 1000 permutation tests at α = 0.05. The percentage of variance explained in the context of a full additive model was calculated using fitqtl.

## 3. Results

### 3.1. Mapping Population Showed Presence/Absence Patterns for Anthocyanin Acylation

**Figure 1:**
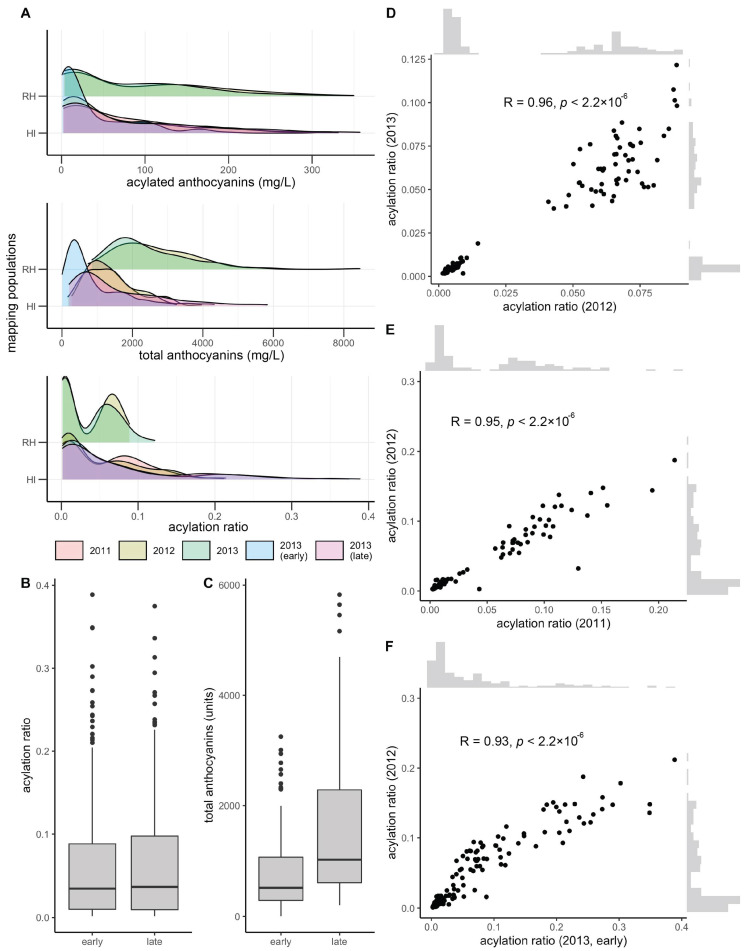
Overview of phenotypic variation. (**A**) Phenotypic variation observed for total anthocyanins, acylated anthocyanins, and acylated ratio (acylated anthocyanins/total anthocyanins). Variation in (**B**) acylation ratio and (**C**) total anthocyanins by harvest time (early vs. late) during 2013 for ‘Horizon’ × Illinois 547-1. Year-to-year Pearson’s correlation for (**D**) *V. rupestris* B38 × ‘Horizon’ and (**E**,**F**) ‘Horizon’ × Illinois 547-1 acylation ratios.

Total and acylated anthocyanins were quantified from a maximum of 112 and 155 vines in the *V. rupestris* B38 × ‘Horizon’ (sampled in 2012 and 2013, at late maturity) and ‘Horizon’ × Illinois 547-1 (sampled in 2011, 2012, and 2013, at late maturity; for the later, sampling at early maturation was also conducted) populations, respectively. The acylation ratio was computed as the ratio of acylated anthocyanins relative to the total anthocyanin concentration. Total anthocyanins and acylated anthocyanins were poorly correlated (R = 0.20, average across years and populations), as were total anthocyanins and acylation ratio (R = −0.25, average across years and populations). However, acylated anthocyanins and acylation ratio were strongly correlated (R = 0.75, average across years and populations). Total anthocyanins and acylated anthocyanins showed a skewed normal distribution, whereas the acylation ratio showed a bimodal distribution, more clearly defined in the *V. rupestris* B38 × ‘Horizon’ population (Figure 1A). Acylation ratio ranged from 0.1% to 12% in *V. rupestris* B38 × ‘Horizon’, and from 0.1% to 39% in ‘Horizon’ × Illinois 547-1. Based on the two sets of phenotypic data collected for the ‘Horizon’ × Illinois 547-1 population, no changes were observed for acylation ratio when comparing early vs. late (*p* = 0.98, *t*-test; Figure 1B); however, total anthocyanin content increased as expected (*p* = 3.74 × 10^−9^; Figure 1C). Pearson’s correlation among years was high (R > 0.93, *p* < 2.2 × 10^−16^) within each population (two years for the *V. rupestris* B38 × ‘Horizon’ population, and 3 years with 2 collection times for the ‘Horizon’ × Illinois 547-1 population; Figure 1D–F). Finally, broad-sense heritability estimates for acylation ratio were 0.95 and 0.84 for the *V. rupestris* B38 × ‘Horizon’ and ‘Horizon’ × Illinois 547-1 populations, respectively. Data analyzed in this study are provided in Supplementary File S1.

### 3.2. Stable QTL for Acylation Ratio across Genetic Backgrounds

**Figure 2:**
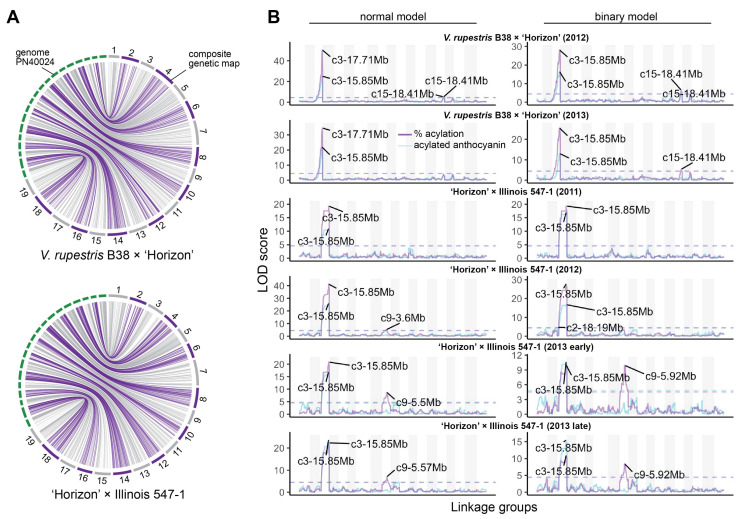
Linkage maps and QTL mapping of anthocyanin acylation. (**A**) Collinearity between physical (PN40024 reference genome) and composite genetic maps for the *V. rupestris* B38 × ‘Horizon’ and ‘Horizon’ × Illinois 547-1 populations. (**B**) Genetic mapping results for each population and evaluation year; QTL mapping was performed using both total acylated anthocyanins (blue line) as well as acylation ratio (purple line); additionally, normal and binary (high vs. low acylation ratio) models were compared for each population/year; dashed lines represent permutation-based significance thresholds.

Composite linkage maps for the *V. rupestris* B38 × ‘Horizon’ and ‘Horizon’ × Illinois 547-1 populations were constructed using rhAmpSeq markers. Of almost 2000 markers successfully amplified in both populations, about 55% were included in the final maps, and the rest were filtered out due to segregation distortion, monomorphism, or missing data. The *V. rupestris* B38 × ‘Horizon’ linkage map included 1092 markers and spanned 1206 cM, with an average gap of 1.16 cM (Table 1). The ‘Horizon’ × Illinois 547-1 linkage map showed similar metrics, including 1171 markers spanning 1182 cM and an average gap length of 1.01 cM. Overall, there was an excellent agreement between the reference genome (PN40024) [26] and the linkage maps of the two populations. Particularly, Spearman’s rank correlation between genetic and physical marker positions was >0.99 (on average across chromosomes) for both populations (Figure 2A).

QTL mapping was performed for each year × population data set using both binary (high vs. low acylation ratios) and continuous (quantitative) single-QTL models. For binary single-QTL models, acylation ratio variables were converted to binary traits (0′s and 1′s), according to the requirements of r/qtl, based on k-means clustering analysis. A large QTL on chromosome 3 (position 14.85–17.95 Mb, based on 1.5-LOD interval) was consistently identified across populations and evaluation years. A stronger signal was observed when using a normal model compared to a binary model (Figure 2B). Additionally, the use of acylation ratio as phenotype instead of the total acylated anthocyanins increased the LOD scores of the identified QTL at chromosome 3. On average, this QTL explained 68.97% and 47.25% of the phenotypic variation (acylation ratio) for the normal and binary single-QTL models, respectively. Significant marker-trait associations on chromosomes 9, 10, and 15 were also discovered; however, these were mostly population-specific (Figure 2B). Although LOD profiles for total acylated anthocyanins and acylation ratio were relatively similar, a QTL on chromosome 9 (position 5.5–5.92 Mb) was revealed only when using acylation ratio on the ‘Horizon’ × Illinois 547-1 population.

### 3.3. Candidate Genes

**Figure 3:**
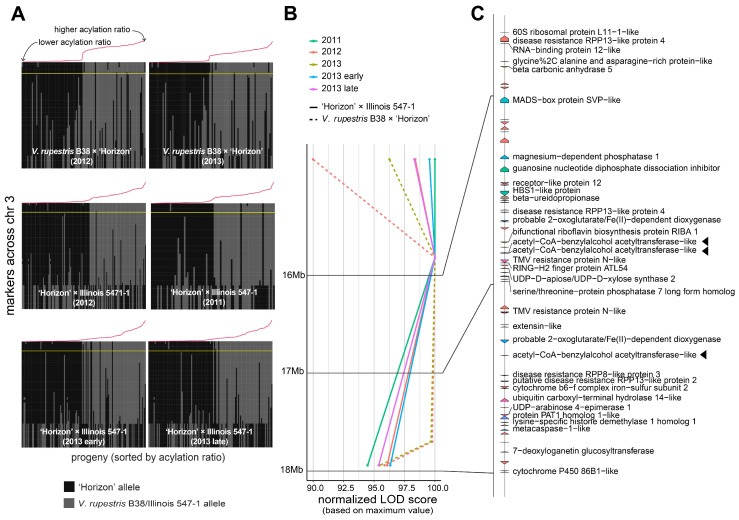
Analysis of the genomic region harboring marker-trait associations on chromosome 3. (**A**) Haplotypes on chromosome 3 for the *V. rupestris* B38 × ‘Horizon’ and ‘Horizon’ × Illinois 547-1 populations. Progenies on each panel are sorted decreasingly, right to left, based on acylation ratio, which is also indicated with a line plot in red color. The largest LOD score per panel is indicated by a horizontal yellow line. (**B**,**C**) LOD score profiles and gene content, respectively, in the 14.85–17.95 Mb interval for all populations and evaluation years. For visualization purposes, LOD scores were normalized to account for differences in the magnitude of the signal; gene labels are shown only for functionally annotated genes. Candidate genes are highlighted with a triangle.

A large QTL on the arm of chromosome 3 was consistently found across different genetic backgrounds and years (Figure 2B). Several genetic markers in that region were highly associated with the percentage of acylation; therefore, a comprehensive analysis of the recombinants in the interval 14.85–17.95 Mb was carried out (Figure 3A). Within the region of interest, there were four markers at positions 14.85, 15.85, 17.71, and 17.95 Mb. These four markers cosegregated almost perfectly with the acylation ratio, except for a few cases (on average, ~3.1 individuals per population), where genotyping errors are a possible explanation. In both populations, high acylation ratios were associated with the ‘Horizon’ genotype.

The 14.85–17.95 Mb region on chromosome 3 of the 12X.v2 PN40024 *V. vinifera* reference genome was inspected on the URGI database (https://urgi.versailles.inra.fr/Species/Vitis/Annotations (accessed on 6 June 2021)) (Figure 3B,C). In total, there were 74 genes, of which 34 had a functional annotation. The genes LOC104878940, LOC100251354, and LOC104878920 (16831069-16831860b, 16834152-16835462b, and 17377464-17378981b, respectively), all encoding an acetyl-CoA-benzylalcohol acetyltransferase-like protein, were in close proximity to the significant markers at positions 15.85 and 17.71 Mb. This location had the largest LOD score across nearly all the evaluated conditions (i.e., genetic backgrounds, year of evaluation, and normal vs. binary QTL models; Figure 3B). Within the 14.85–17.95 Mb interval, version 4 of the PN40024 genome assembly (https://integrape.eu/resources/genes-genomes/genome-accessions/ (accessed on 26 November 2021)) lists not three but four genes encoding an acetyl-CoA-benzylalcohol acetyltransferase-like protein, which could be explored in further fine-mapping studies.

## 4. Discussion

Anthocyanins serve a dual role in the food industry as natural pigments and as health-promoting dietary constituents purported to have multiple beneficial properties [9,10,11]. Acylation of anthocyanins affects both attributes yet with contrasting consequences. Acylation increases the color stability of anthocyanins, reducing their sensitivity to pH change, sulfite bleaching, and thermal degradation [4]. Conversely, compared to non-acylated forms, the bioavailability of acylated anthocyanins is substantially lower, consequently limiting their potential benefit following dietary intake [7,8]. As a result, the desired ratio of acylation may vary depending on the purpose and required properties of the anthocyanins. For instance, a high acylation rate may be regarded as advantageous in the case of food colorants but undesirable in the case of dietary supplements. Thus, molecular markers for acylation provide a valuable tool for grape breeders to shift the ratio of acylation in either direction.

In this study, the portion of acylated anthocyanins that existed in acylated form (“acylated anthocyanin ratio”; 0.1%–39%) followed a clear binomial distribution within each population (*V. rupestris* B38 × ‘Horizon’ and ‘Horizon’ × Illinois 547-1). These ranges for acylation ratios were within previously observed ranges in both *V*. *vinifera* and wild *Vitis* accessions [5,6]. Acylated anthocyanins and acylation ratio were highly correlated, while total anthocyanins and acylation ratio were uncorrelated. This was likely a consequence of the large range in acylated anthocyanin concentrations within a population as compared to total anthocyanins (3 vs. 1 orders of magnitude, respectively).

Using highly transferable rhAmpSeq markers, we mapped a significant association between the anthocyanin acylation ratio and a genomic region harboring three copies of a gene encoding acetyl-CoA-benzylalcohol acetyltransferase-like protein. Previously, a weaker QTL (based on a single year of data from the ‘Horizon’ × Illinois 547-1 population genotyped with GBS) located nearby was detected using total acylated anthocyanins, emphasizing the advantage in normalizing acylated anthocyanin concentration against total anthocyanins [28]. Acetyl-CoA-benzylalcohol acetyltransferase-like proteins are part of the enzyme super-class of BAHD transferases, which can catalyze acylation reactions from a broad range of anthocyanins and acyl-donating compounds [13]. The BAHD superfamily includes the anthocyanin 3-*O*-glucoside-6-*O*-acyltransferase (*3AT*) described by Rinaldo et al. [13], in which a nonsense mutation prevents the production of acylated anthocyanins in Pinot noir. Moreover, transcriptomic studies have also shown that *3AT* expression increases in the skins of most red berries (but not white) during ripening in both *V. vinifera* [13,14] and wild *Vitis* [29].

Mapping population studies in other plant families, e.g., maize, have also reported that anthocyanin acyltransferases are important for controlling acylation ratios [30]. Thus, *3AT* is a likely candidate for controlling acylation ratios in grapes. Notably, both extreme alleles producing high acylation in the *V. rupestris* B38 × ‘Horizon’ and ‘Horizon’ × Illinois 547-1 populations came from ‘Horizon’, which produces white fruit. This cultivar resulted from a cross between ‘Seyval blanc’ and ‘Schuyler’, and the latter was a hybrid of ‘Primitivo’ (a red wine variety) and ‘Ontario’ (white-fruited). White-fruited grapes result from loss-of-function mutations of VvMYBA protein [31,32], a major regulator of anthocyanin accumulation, which acts upstream and regulates the expression of *Vv3AT* [13]. Therefore, as indicated in this case, while phenotypically masked by *VvMYBA* loss-of-function, white-fruited genotypes can form a valuable genetic source of highly functional Vv3AT alleles.

Interestingly, two previous studies on *V. vinifera* mapping populations did not detect major QTLs for acylation ratio, nor a QTL on LG 3 associated with *Vv3AT*. A thorough study of anthocyanin profiles in a ‘Syrah’ × Pinot noir population yielded only minor QTLs for anthocyanin acylation on LGs 3, 7, 8, 12, and 18. The authors speculate that their inability to detect a major QTL may be due to low segregation of the acylated ratio trait [15]. Production of non-functional copies of *Vv3AT* in Pinot noir [13] may reflect homozygosity for this nonsense mutation, and if ‘Syrah’ is homozygous for the two functional *Vv3AT* copies, the resulting population would be heterozygous. A separate study on an F1 *V. vinifera* mapping population (‘Red Globe’ × ‘Muscat Hamburg’) also identified weak QTLs for acylation ratio [33]. Again, this study may have been limited by the low amount of trait variation within the population, as the distribution was unimodal, with most acylation ratios falling between 20% and 40%. These earlier reports identified several other candidate genes, including other anthocyanin acyltransferases (*VvAT1*, *VvAT9*) as well as genes involved in ion transport. We did not detect these same QTL, although we did detect a minor QTL in one year and one population (2013, ‘Horizon’ × Illinois 547-1) on Chr 9. The interval for this QTL contains a 5-O-glucosyltransferase (*5GT*) responsible for the production of anthocyanin 3,5-diglucosides in wild *Vitis* species. However, the relationship between diglucosides and acylated anthocyanins is unclear.

The previous report with one harvest year on ‘Horizon’ × Illinois 547-1 used GBS to develop a haploblock panel of thirteen amplicon sequencing SNP markers to predict acylated ratios in *V. rupestris* B38 × ‘Horizon’ [28]. However, because the transferability of SNP-based markers has been low in high-diversity taxa such as *Vitis* spp., these markers have not been widely implemented. Highly transferable molecular markers are essential for the implementation of marker-assisted selection strategies, especially in grapes where the use of wild relative species in breeding programs is fairly common [19,34]. Unlike markers derived from skim sequencing platforms, PCR-based markers such as rhAmpSeq used here to map acylation ratio can be more readily implemented in current grape breeding strategies [19] since these have been tested for transferability, cover the whole genome, and have the capability to differentiate germplasm. The high phenotypic variance explained by the associated markers on chromosome 3 will make these rhAmpSeq markers particularly efficient, once properly validated, when predicting high vs. low acylation ratio in *Vitis* species.

## Figures and Tables

**Table 1:** Features of the *V. rupestris* B38 × ‘Horizon’ and ‘Horizon’ × Illinois 547-1 sex-averaged linkage maps.

Chr	*V. rupestris* B38 × ‘Horizon’					‘Horizon’ × Illinois 547-1				
	Markers	Size (cM)	Average Gap (cM)	Maximum Gap (cM)	Recomb. Rate (cM/Mb)	Markers	Size (cM)	Average Gap (cM)	Maximum Gap (cM)	Recomb. Rate (cM/Mb)
1	42	37.30	0.91	4.83	0.98	85	62.34	0.72	4.97	1.99
2	40	62.51	1.60	12.0	2.84	50	56.82	1.16	12.0	4.31
3	42	51.08	1.25	5.13	3.33	40	55.70	1.43	18.5	3.70
4	64	75.39	1.20	6.35	0.56	68	54.52	0.81	5.46	1.88
5	81	61.89	0.77	4.16	1.97	90	49.94	0.56	3.13	0.93
6	56	66.27	1.20	5.43	3.08	65	49.49	0.77	6.01	0.11
7	74	85.58	1.17	8.31	1.69	93	84.12	0.91	9.08	2.94
8	76	67.94	0.91	5.82	1.79	85	66.60	0.79	4.40	2.00
9	47	53.88	1.17	8.64	1.86	46	49.14	1.09	8.80	5.00
10	51	62.53	1.25	6.42	2.35	45	51.09	1.16	8.30	1.91
11	47	62.28	1.35	6.53	3.96	47	52.77	1.15	5.56	5.25
12	59	60.65	1.05	9.31	2.27	55	49.23	0.91	3.62	4.03
13	67	65.30	0.99	10.9	1.73	67	56.79	0.86	5.76	0.94
14	84	68.54	0.83	4.60	2.24	78	60.07	0.78	3.58	2.46
15	43	55.54	1.32	8.24	4.05	31	53.02	1.77	8.51	5.03
16	47	59.24	1.29	7.20	2.62	42	50.41	1.23	5.32	2.10
17	53	67.88	1.31	10.7	1.19	52	53.58	1.05	6.89	1.49
18	67	82.16	1.24	8.18	1.27	76	69.94	0.93	6.58	3.24
19	52	60.62	1.19	6.22	2.80	56	56.59	1.03	8.10	2.88
**Total**	**1092**	**1206.58**	**1.16**	**7.31**	**2.24**	**1171**	**1082.16**	**1.01**	**7.08**	**2.75**

## Data Availability

Haplotype sequences of the genetic markers, sex-averaged genetic maps, and phenotyping data sets are given as supplementary files. Perl scripts used for identification of haplotype variants and conversion to a variant calling format (VCF) are available at: https://github.com/avinashkarn/analyze_amplicon/blob/master/analyze_amplicon.pl (accessed on 2 April 2021) and https://github.com/avinashkarn/analyze_amplicon/blob/master/haplotype_to_VCF.pl (accessed on 2 April 2021), respectively.

## Supplementary Materials

The following are available online at https://www.mdpi.com/article/10.3390/genes12121962/s1, Supplementary File S1: Best linear unbiased estimators for total anthocyanins, acylated anthocyanins, and acylation ratio in the *V. rupestris* B38 × ‘Horizon’ and ‘Horizon’ × Illinois 547-1 populations., Supplementary Files S2 and S3: Genotyping QC for ‘Horizon’ × Illinois 547-1 and *V. rupestris* B38 × ‘Horizon’ families, respectively., Supplementary Files S4 and S5: Four-way cross-genetic maps for ‘Horizon’ × Illinois 547-1 and *V. rupestris* B38 × ‘Horizon’ families, respectively.
