# Supplementary figures and images for: The Genetic Basis of Anthocyanin Acylation in North American Grapes (Vitis spp.)
Source: Genes (Basel). 2021 Dec 9;12(12):1962. doi: 10.3390/genes12121962 (PMC8701791; doi:10.3390/genes12121962)

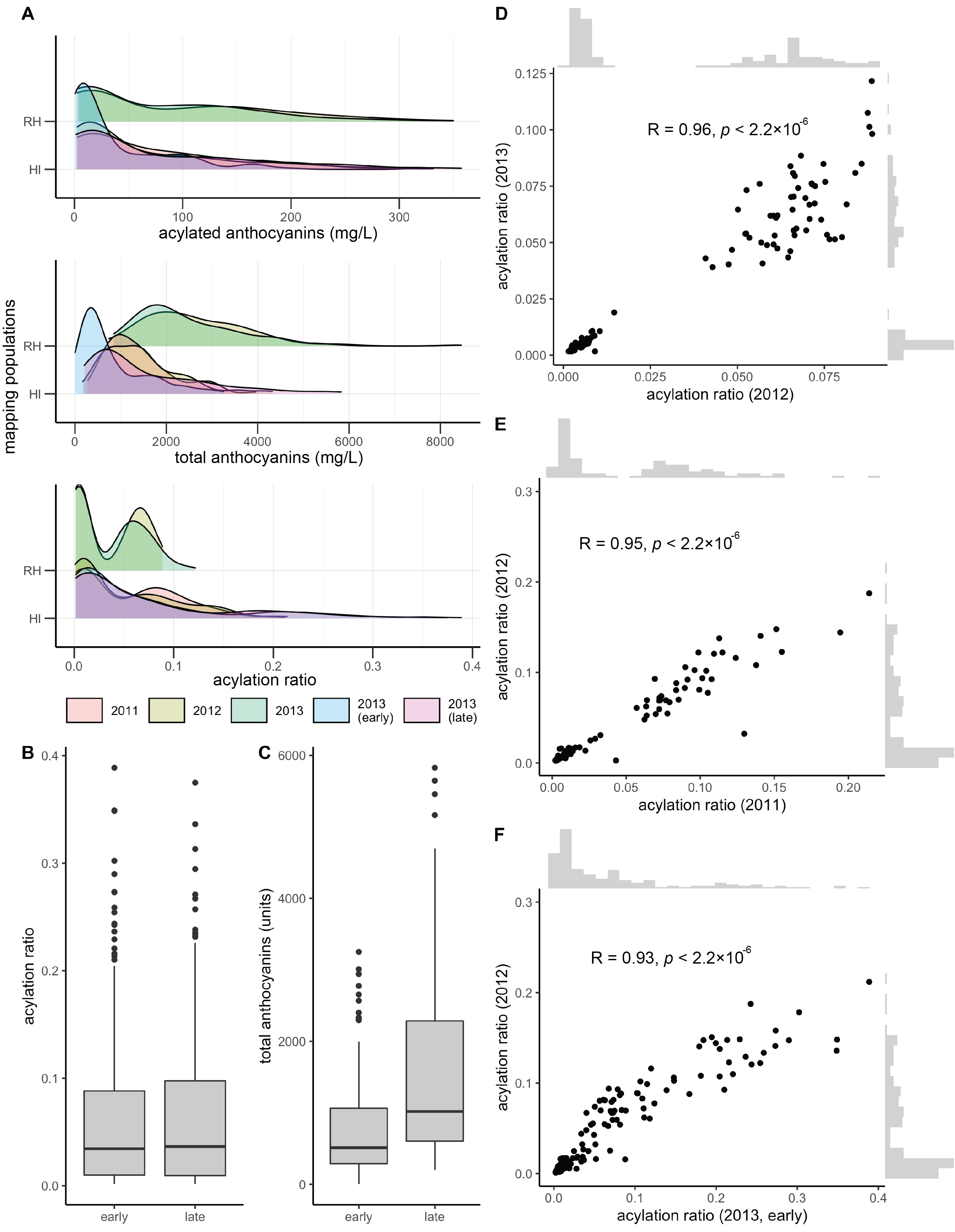

Supplement: Supplementary file 1 [file genes-12-01962-s001.zip › Fig 1.png]

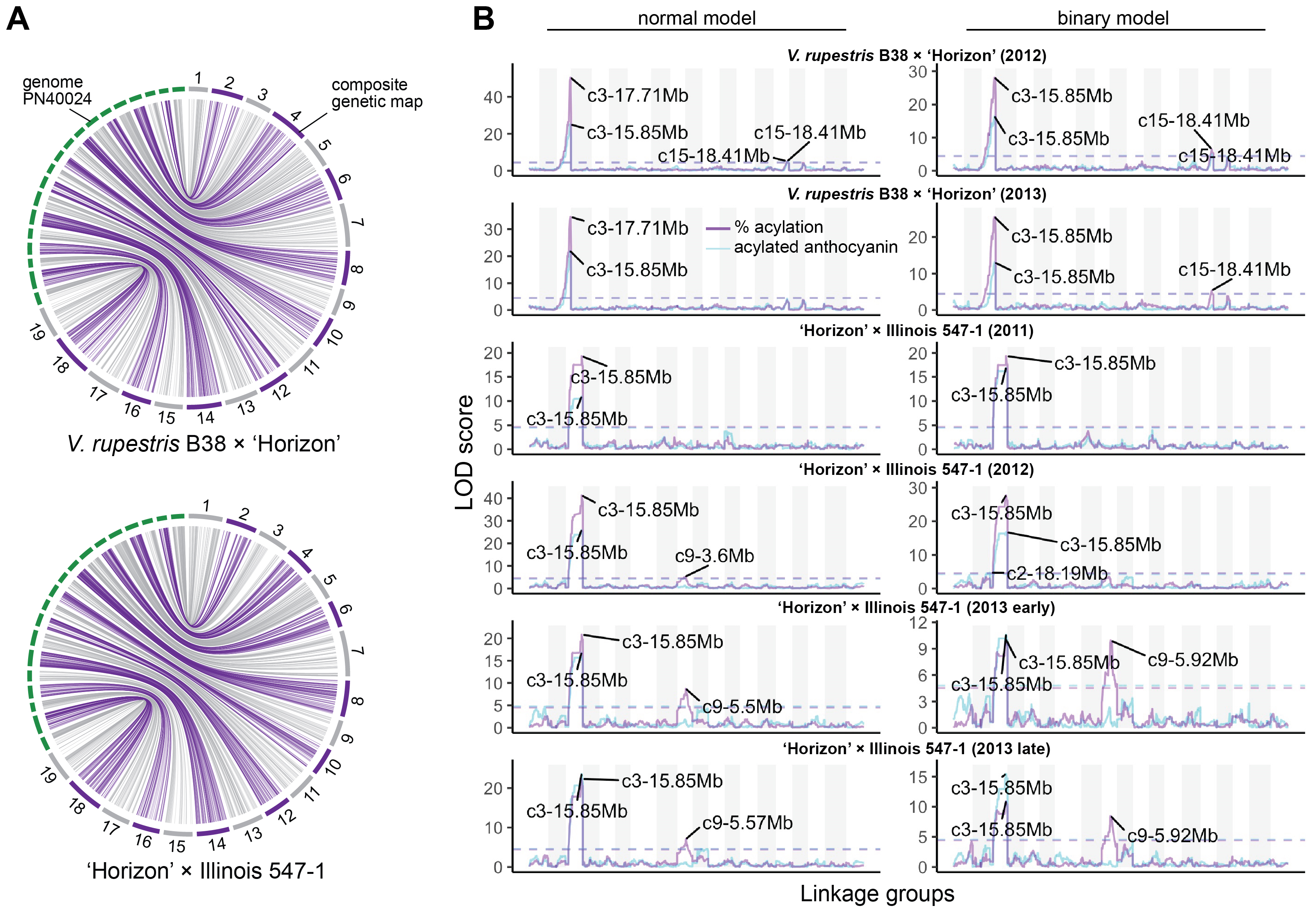

Supplement: Supplementary file 1 [file genes-12-01962-s001.zip › fig 2.png]

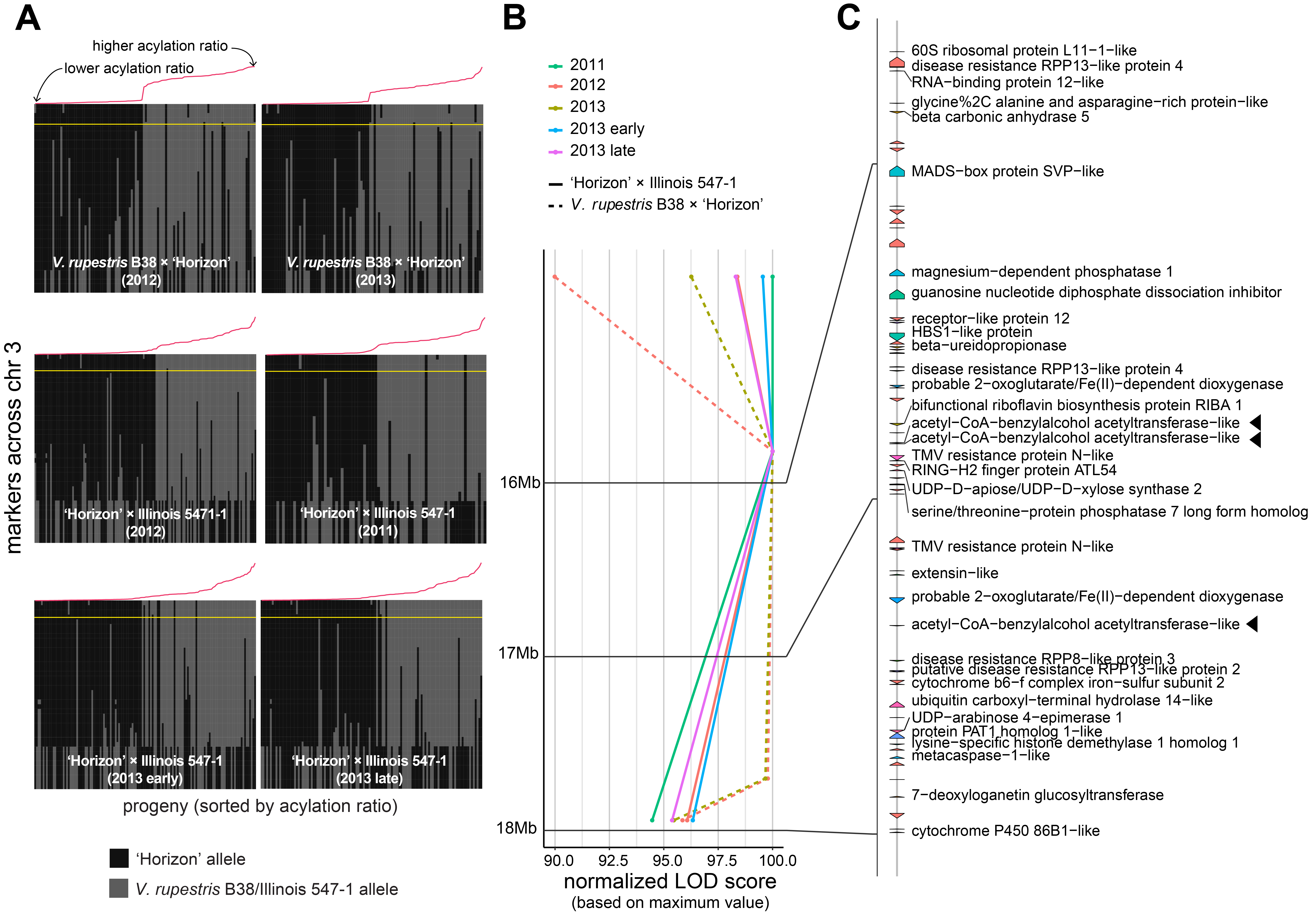

Supplement: Supplementary file 1 [file genes-12-01962-s001.zip › Fig 3.jpg.png]
